# Comparison Between In‐Hospital and Community‐Onset Stroke Treated With Endovascular Thrombectomy: A Propensity Score–Matched Cohort Study

**DOI:** 10.1161/SVIN.122.000816

**Published:** 2023-04-25

**Authors:** Permesh Singh Dhillon, Emma Soo, Waleed Butt, Thanh N. Nguyen, Emma Barrett, Anna Podlasek, Norman McConachie, Robert Lenthall, Sujit Nair, Luqman Malik, Chesvin Cheema, Pervinder Bhogal, Hegoda Levansri Dilrukshan Makalanda, Martin A. James, Robert A. Dineen, Timothy J. England

**Affiliations:** ^1^ Interventional Neuroradiology Nottingham University Hospitals NHS Trust Nottingham UK; ^2^ NIHR Nottingham Biomedical Research Centre Nottingham UK; ^3^ Radiological Sciences, Mental Health & Clinical Neuroscience, School of Medicine University of Nottingham Nottingham UK; ^4^ Interventional Neuroradiology University Hospitals Birmingham NHS Trust UK; ^5^ Department of Neurology Boston Medical Center Boston MA; ^6^ Department of Radiology, Boston Medical Center Boston University School of Medicine Boston MA; ^7^ Department of Research and Innovation (Medical Statistics) Manchester University NHS Foundation Trust Manchester UK; ^8^ Centre for Biostatistics, Faculty of Biology Medicine and Health University of Manchester Manchester UK; ^9^ Tayside Innovation Medtech Ecosystem (TIME) University of Dundee UK; ^10^ Faculty of Medicine University of Queensland Queensland Australia; ^11^ Interventional Neuroradiology Barts Health NHS Trust London UK; ^12^ Exeter Medical School University of Exeter Exeter UK; ^13^ Stroke Royal Devon and Exeter NHS Foundation Trust Exeter UK; ^14^ Sentinel Stroke National Audit Programme King's College London UK; ^15^ Stroke Trials Unit, Mental Health & Clinical Neuroscience, School of Medicine University of Nottingham Nottingham UK; ^16^ Stroke University Hospitals of Derby and Burton NHS Foundation Trust UK

**Keywords:** hospital, ischaemia, occlusion, stroke, thrombectomy

## Abstract

**Background:**

Patients with acute ischemic stroke onset during hospital admission often have concurrent illnesses, increased underlying comorbidities and are often associated with a delayed recognition of stroke onset, compared with patients with stroke onset in the community (community‐onset stroke [COS]). Endovascular thrombectomy (EVT) for large‐vessel occlusion in acute ischemic stroke has been proven to be effective, though the safety and feasibility of EVT among patients with in‐hospital stroke (IHS) onset remains undetermined. We aim to compare the workflow and clinical outcomes for patients undergoing EVT following IHS onset and COS.

**Methods:**

Using data from a national stroke registry, we used propensity score‐matched individual‐level data of patients who underwent EVT, following IHS and COS, between October 2015 and March 2020. Univariate analysis was performed to assess the procedural, functional, and safety outcomes.

**Results:**

We included 4353 patients (COS, 4104 [249 after propensity score matching]; IHS, 249 [249 after propensity score matching]). Compared with COS, patients with IHS had similar modified Rankin Scale on discharge (odds ratio [OR], 0.98 [95% CI, 0.72–1.34]; *P*=0.96) and at 6 months (OR, 1.25 [95% CI, 0.71–2.24]; *P*=0.48). No significant difference in achieving good functional outcome (modified Rankin Scale ≤ 2 at discharge; 31.3% [IHS] versus 29.3% [COS]; OR,=1.10 [95% CI 0.74–1.60]; *P*=0.61), successful reperfusion (modified Thrombolysis in Cerebral Infarction score of 2b–3), *P*=0.82; or safety outcomes of symptomatic intracranial hemorrhage (*P*=0.64) and in‐hospital mortality (*P*=0.26) were demonstrated. Shorter time interval from stroke onset to imaging in the IHS group (IHS, 80±88 versus COS, 216±292 minutes) was observed. The imaging‐to‐arterial‐puncture time was not significantly different between the groups (IHS, 160±140 versus COS, 162±184 minutes; *P*=0.85).

**Conclusions:**

EVT in patients with IHS is safe and feasible, with comparable functional and safety outcomes to patients with COS, in this national stroke registry. Continued efforts are required to improve the inpatient stroke workflow in recognizing stroke symptoms and initiating reperfusion treatment for eligible patients with IHS.

**Nonstandard Abbreviations and Acronyms**

COS: community‐onset stroke
ECASS II: European Collaborative Acute Stroke Study II
EVT: endovascular thrombectomy
IHS: in‐hospital stroke
mRS: modified Rankin Scale
NIHSS: National Institutes of Health Stroke Scale
sICH: symptomatic intracranial hemorrhage
PSM: propensity score matching
SSNAP: Sentinel Stroke National Audit Programme

**Clinical Perspective**

- We sought to investigate the workflow, safety and functional outcomes following endovascular thrombectomy in patients with in‐hospital stroke onset compared to patients with community onset stroke in a national stroke registry.
- Patients with in‐hospital stroke onset and treated with endovascular thrombectomy had similar rates of functional outcome, symptomatic intracranial hemorrhage, and mortality, compared to those with community onset stroke.
- Our findings suggest that endovascular thrombectomy appears safe and feasible in patients with in‐hospital stroke onset.

In‐hospital onset of acute ischemic stroke accounts for 2.2% to 17% of all strokes, and a proportion of this is due to large‐vessel occlusion.^1^, ^2^, ^3^ Compared with community‐onset stroke (COS), patients with in‐hospital stroke (IHS) onset often have concurrent illnesses and increased underlying comorbidities and risk factors, including cardiovascular disease, malignancy, and invasive procedures.^4^ These factors may affect the functional reserve of patients and the potential risk‐benefit ratio of treatment. Delays in stroke recognition and imaging, as well as lower rates of intravenous thrombolysis due to contraindications such as concurrent antithrombotic use or recent surgery have also been reported, contributing to the lower rates of functional independence and higher rates of mortality in the IHS cohort.^5^

Endovascular thrombectomy (EVT) for large‐vessel occlusion in acute ischemic stroke has been proven to be effective when initiated up to 24 hours of stroke onset and allows a broader inclusion of patients who may have been ineligible for intravenous thrombolysis.^6^, ^7^ However, there is a paucity of data on the characteristics and clinical outcomes in patients with IHS treated with EVT. We hypothesize that EVT‐treated patients with IHS onset are more likely to have a poor clinical outcome compared with those with COS. Hence, we sought to investigate the workflow, safety, and functional outcomes following EVT in patients with IHS‐onset compared with patients with COS in a national stroke registry.

## Methods

### Ethics

The SSNAP (Sentinel Stroke National Audit Programme) registry has permission to collect patient data without explicit patient consent, granted by the Confidentiality Advisory Group of the National Health Service Health Research Authority under Section 251. Pseudonymized/deidentified data use was approved by the Healthcare Quality Improvement Partnership Data Access Request Group (Ref: HQIP366). Additional ethical approval was not sought or required for this study (Ref: 21‐037C). Data access requests should be directed to SSNAP as the data provider and Healthcare Quality Improvement Partnership as the data controller.

### Data Source and Study Design

We performed a cohort study using prospectively collected data from patients enrolled in the SSNAP registry according to the Strengthening the Reporting of Observational Studies in Epidemiology guidelines.^8^ SSNAP is a national stroke registry that includes all hospitals admitting patients with acute stroke in England, Wales, and Northern Ireland (covering 92% of the population of the United Kingdom).^9^ Overall case ascertainment in SSNAP is estimated to be >90% of all acute stroke admissions.^9^ Patient data, which include demographic and clinical characteristics, treatments, and outcomes, are submitted prospectively by clinical teams using a secure web‐based case report form with real‐time data validation checks to ensure data quality, from the time of admission up to 6 months after stroke.

Pseudonymized individual‐level data of adult patients (≥18 years) presenting with acute ischemic stroke who received EVT between October 1, 2015 (inception of the EVT section of SSNAP) and March 31, 2020, in England and Wales were included. Patients were dichotomized according to the location of the patient during the onset of stroke to (1) in‐hospital and (2) in the community. Patients with missing discharge modified Rankin Scale (mRS) data were excluded.^10^ The selection of EVT‐eligible patients was at the discretion of the practitioners on the basis of each institution's protocol. No specific limits were applied to the clinical inclusion criteria, including age, prestroke disability (mRS), and baseline stroke severity on the National Institutes of Health Stroke Scale (NIHSS). Data on the parenchymal imaging findings and clot location were not available in the registry.

### Outcome Measures

The main functional outcome was assessed with the mRS score at hospital discharge, ranging from 0 (no symptoms) to 5 (severe disability/bedridden) and 6 (death).^11^ Other functional outcomes were the mRS score at 6 months, good (mRS ≤2 or equivalent to the prestroke mRS) or excellent (mRS ≤1 or equivalent to the prestroke mRS) functional outcome at hospital discharge and at 6 months, early neurological improvement (NIHSS decrease ≥4 between admission and 24 hours or NIHSS 0–1 at 24 hours), and early neurological deterioration (24‐hour NIHSS increase ≥4 from baseline). Procedural outcomes were successful reperfusion (modified thrombolysis in cerebral infarction score of 2b–3) and complete reperfusion (modified Thrombolysis in Cerebral Infarction score of 3) at the end of EVT.

Safety outcomes were in‐hospital mortality, any type of intracranial hemorrhage and symptomatic intracranial hemorrhage (sICH) defined according to the ECASS II (European Collaborative Acute Stroke Study II)^12^ as any intracranial hemorrhage with an increase of the NIHSS score of ≥4 within 24 hours or death. Workflow time metrics were stroke onset to neuroimaging, imaging to arterial puncture, and total procedural time (defined as arterial puncture to final reperfusion/angiographic run). Functional outcome measure (mRS) was assessed by a member of the stroke team/physician at discharge and during a routinely scheduled clinical visit at 6 months or by a specialist nurse during a follow‐up telephone interview if the patient was unable to attend.

### Statistical Analysis

Study characteristics were summarized using descriptive statistics for patient demographics, clinical characteristics and comorbidities, EVT technique, and time metrics. Normality of data distribution was assessed using the Shapiro–Wilk test. Continuous variables were expressed as means and SD, and categorical variables were expressed as frequencies or percentages. Comparisons of baseline variables were made using the chi‐square or Student's *t*‐test where applicable.

Propensity score matching (PSM), a method of decreasing potential bias in cohorts with multiple confounders, was conducted with a 1:1 matching of the logit of the propensity score using the nearest‐neighbor (Greedy type) matching and 0.2 caliper width.^13^ The matching was performed without replacement, and unpaired patients not meeting the matching criteria were excluded. Each PSM‐derived pair was created using the R package MatchIt (R Foundation for Statistical Computing, Vienna, Austria). The key variables accounted for in the PSM were age (5‐year age bands from <60 to >90 years), sex, baseline stroke severity (NIHSS), prestroke functional status (mRS), time to imaging, and prior administration of intravenous thrombolysis.

Since the major confounders were accounted for using PSM, univariate analyses of the outcome measures used ordinal logistic regression for the full‐scale mRS; binary regression analysis for dichotomized mRS scores (good functional outcome mRS ≤2, and excellent functional outcome mRS ≤1), early neurological improvement, early neurological deterioration, successful reperfusion modified Thrombolysis in Cerebral Infarction 2b–3, complete reperfusion modified Thrombolysis in Cerebral Infarction 3, any intracranial hemorrhage, sICH, and death. Analyses of binary and ordinal outcomes were expressed as an odds ratio (OR) with a 95% CI. Missing outcome data were not imputed. A 2‐tailed *P* value of <0.05 was considered statistically significant. All analyses were conducted using StataSE 17.1 (StataCorp, College Station, TX) and R 4.1.0.

## Results

### Characteristics of Study Population

**Figure 1:**
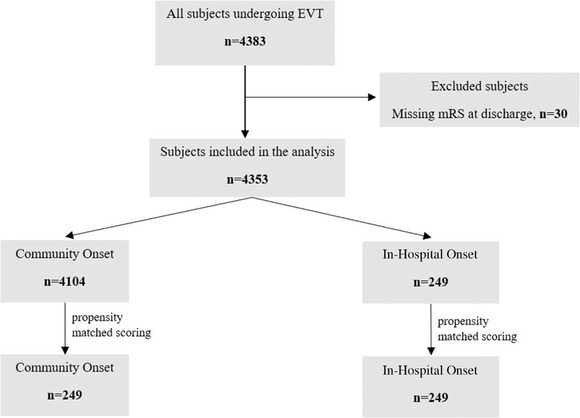
**Flow chart of the patient inclusion, exclusion, and outcome data for endovascular thrombectomy treated patients** **with stroke onset in‐hospital (IHS) and stroke onset in the community (COS)**. COS indicates community‐onset stroke; EVT, endovascular thrombectomy; IHS, in‐hospital stroke; mRS, modified Rankin Scale; and n, number of events.

**Figure 2:**
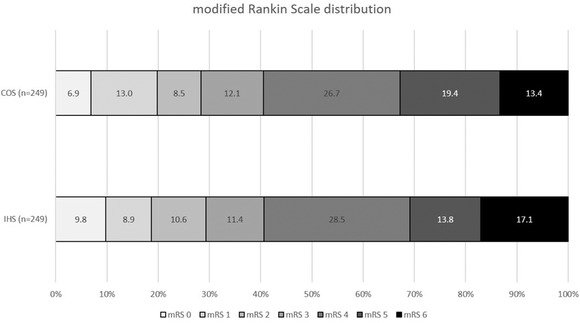
**Distribution of the propensity scores of patients who underwent endovascular thrombectomy following** **IHS and COS**. Patients were matched 1:1 for age, sex, baseline NIHSS, prestroke disability, time to imaging, and use of intravenous thrombolysis. COS indicates community‐onset stroke; IHS, in‐hospital stroke; mRS, modified Rankin Scale; and NIHSS, National Institutes of Health Stroke Scale.

A total of 4383 patients admitted to 123 hospitals, of which 25 were EVT‐capable neuroscience centers, underwent EVT during the study period. Of these, 30 patients with a lack of data on the mRS score at discharge were excluded (Figure 1). A total of 4104 patients with COS and 249 (5.7%) patients with IHS were included. In the total cohort, before PSM, patients in the IHS group had a higher prestroke disability (mRS) (1±1 versus 0±1; *P*=0.002) and a significantly shorter time interval from stroke onset to imaging compared with the COS group (80±88 versus 216±292 minutes; *P*=0.001). No significant differences were observed in the remaining baseline characteristics or comorbidities. After PSM, there were 249 patients with IHS and COS, respectively, and all matched baseline characteristics were similar (Table 1). The distribution of propensity scores of patients across both groups is presented in Figure 2.

**Table 1:** Table of Characteristics Comparing Patients Treated With Endovascular Thrombectomy Following IHS and COS Before and After PSM.

Feature	Before PSM			After PSM*		
	COS, n (%) or mean±SD	IHS, n (%) or mean±SD	*P* value	COS, n (%) or mean±SD	IHS, n (%) or mean±SD	*P* value
Sociodemographics						
Sample size	4104	249		249	249	
Sex, male	2245 (55)	128 (51)	0.21	128 (51)	128 (51)	1.00
Age, <60 y	3388	64 (26)	0.99	59 (24)	64 (26)	0.82
60–69	1468 (36)	51 (20)		46 (18)	51 (20)	
70–79	1028 (25)	74 (30)		84 (33)	74 (30)	
80–89	1161 (28)	51 (20)		56 (22)	51 (20)	
>90 y	1113	94		42	94	
Baseline characteristics						
NIHSS on admission	17±7	16±7	0.50	16±6	16±7	0.96
Prestroke disability (mRS)	0±1	1±1	0.002	1 ±1	1±1	0.96
Intravenous thrombolysis	2434 (59)	136 (55)	0.092	134 (54)	136 (55)	0.86
Stent retriever	651 (18)	3215	0.22	38 (18)	3215	0.53
Contact aspiration	1226 (33)	74 (34)	0.97	85 (39)	74 (34)	0.29
Combined stent retriever and contact aspiration	1793 (49)	111 (51)	0.61	93 (43)	111 (51)	0.10
Proximal balloon flow arrest	918 (22)	55 (22)	0.97	54 (22)	55 (22)	0.91
Local anesthesia	1400 (34)	93 (38)	0.31	90 (36)	93 (38)	0.78
Conscious sedation	54 713	3313	0.99	4016	3313	0.38
General anesthesia	2145 (52)	121 (49)	0.26	117 (47)	121 (49)	0.72
Comorbidities						
Hypertension	1953 (48)	123 (49)	0.49	125 (50)	123 (49)	0.86
Diabetes	57 414	3012	0.42	3313	3012	0.69
Atrial fibrillation	893 (22)	53 (21)	0.91	58 (23)	53 (21)	0.59
Congestive heart failure	2105	114	0.65	94	114	0.65
Prior stroke/transient ischemic attack	62 815	4116	0.58	4217	4116	0.90
Time metrics (minutes)						
Onset to brain imaging	216±292	80±88	<0.001	85±90	80±88	0.50
Brain imaging to arterial puncture	162±184	160±140	0.85	154±165	160±140	0.71
Groin puncture to end of procedure	57±38	56±38	0.46	58±41	56±38	0.41

COS indicates community‐onset stroke; IHS, in‐hospital stroke; mRS, modified Rankin Scale; NIHSS, National Institutes Stroke Severity; and PSM, propensity score matching.

* Propensity score matched 1:1 for age, sex, baseline NIHSS, prestroke disability, time‐to‐imaging, and use of intravenous thrombolysis.

### Outcomes (After Propensity Score Matching)

**Figure 3:**
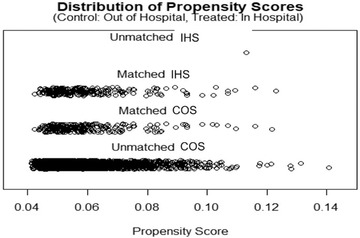
**Distribution of the modified Rankin scale (0 [no disability] to 5 [severe disability] and 6 [death]) at discharge comparing endovascular thrombectomy treated patients with IHS and COS after propensity score matching**. COS indicates community‐onset stroke; and IHS, in‐hospital stroke.

When compared with EVT treatment in patients with COS, patients with IHS had similar mRS scores at discharge (ordinal shift: common OR=0.98 [95% CI, 0.72–1.34]; *P*=0.96; Figure 3 , Table 2). No difference was observed in the odds of achieving good functional outcome (mRS ≤2 at discharge; 31.3% [IHS] versus 29.3% [COS], OR, 1.10 [95% CI, 0.74–1.60]; *P*=0.61), successful reperfusion (IHS, 79.9% versus COS, 79.1%; *P*=0.82), sICH (IHS, 1.9% versus COS, 2.6%; *P*=0.64), or in‐hospital mortality (IHS, 16.9% versus COS, 13.3%; *P*=0.26) across both groups (Table 2).

**Table 2:** Table of Outcomes Comparing Patients Treated With Endovascular Thrombectomy Following IHS and COS After PSM

Outcome measures	Community onset n/N (%) or median (IQR)	In‐hospital onset n/N (%) or median (IQR)	COS vs IHS	
			OR (95%CI)*	*P* value
mRS at discharge (ordinal)	4 (2–5)	4 (2–5)	0.98 (0.72–1.34)	0.96
mRS ≤1	49/249 (19.7)	46/249 (18.5)	0.94 (0.58–1.47)	0.76
mRS ≤2	73/249 (29.3)	78/249 (31.3)	1.10 (0.74–1.60)	0.61
mRS at 6 mo (ordinal)	2 (1–3)	2 (1–4)	1.25 (0.71–2.24)	0.48
mRS ≤2	49 /170 (28.8)	38 /168 (22.6)	0.72 (0.39–1.37)	0.29
TICI2b–3	197/249 (79.1)	199/249 (79.9)	1.04 (0.67–1.61)	0.82
TICI3	125/249 (50.2)	112/249 (45.0)	0.82 (0.58–1.18)	0.24
ENI	135/230 (58.7)	137/234 (58.5)	0.99 (0.69–1.44)	0.97
END	25/230 (10.9)	21/234 (9.0)	0.81 (0.44–1.49)	0.51
Any ICH	26/168 (15.5)	23/175 (13.1)	0.85 (0.48–1.52)	0.55
sICH	4/154 (2.6)	3/162 (1.9)	0.70 (0.17–3.22)	0.64
In‐hospital mortality	33/249 (13.3)	42/249 (16.9)	0.35 (0.82–2.20)	0.26

COS indicates community‐onset stroke; END, early neurological deterioration (NIHSS worsening by ≥4); ENI, early neurological improvement (NIHSS improvement by ≥4); IHS, in‐hospital stroke; IQR, interquartile range; mRS, modified Rankin scale; NIHSS, National Institutes Stroke Severity; OR, odds ratio; PSM, propensity score matching; sICH, symptomatic intracranial hemorrhage; and TICI, Thrombolysis in Cerebral Infarction.

* Propensity score matched 1:1 for age, sex, baseline NIHSS, prestroke disability, time to imaging, and use of intravenous thrombolysis.

## Discussion

The findings in our study provide real‐world data into the workflow and functional and safety outcomes of EVT treatment in patients with IHS onset. There was a significantly shorter interval between stroke onset to neuroimaging in patients with IHS while the neuroimaging‐to‐arterial‐puncture interval was marginally longer compared with patients with COS. EVT performed in patients with IHS accounted for 5.7% of the total cohort and was associated with similar rates of functional outcome at discharge and at 6 months, sICH, and in‐hospital mortality, compared with those treated following COS. Overall, this suggests that performing EVT in patients with IHS appears safe and equally effective, achieving similar rates of functional independence (mRS ≤2), as those with COS.

Previous retrospective studies of modest sample sizes have attempted to assess the feasibility of EVT among patients with IHS compared with those with COS, yielding conflicting results. While some single‐center institutional case series have shown equivalent functional outcomes^14^, ^15^, ^16^ and shorter stroke onset‐to‐imaging times in the IHS group,^15^, ^16^ findings from a separate national US registry demonstrated longer stroke presentation‐to‐imaging times, poorer functional outcomes at hospital discharge, and higher in‐hospital mortality rates among the IHS cohort.^5^ The findings in our study seem plausible as both patient cohorts shared similar baseline characteristics including stroke severity (NIHSS) and comorbidities, and similar onset‐to‐imaging times after PSM. It is conceivable that patients with IHS likely suffer from other comorbidities and concurrent illnesses such as underlying malignancy or sepsis, which may negatively influence the functional outcome and in‐hospital mortality rates.^16^ However, these additional data were not available in this registry and hence could not be evaluated in further detail. The use of intravenous thrombolysis was also marginally lower among patients with IHS compared with COS, in line with previous studies^1^ and is likely due to the higher likelihood of potential contraindications for its use following recent procedures or concurrent antithrombotic use.

Although a shorter time interval from stroke onset to neuroimaging was observed in the IHS cohort (80±88 versus 216±292 minutes) compared with the COS group before PSM, further improvement in the workflow of stroke recognition, early mobilization of inpatients for neuroimaging, and prompt referral to the dedicated stroke and neurointerventional teams should be implemented, particularly considering that these patients are already present in a hospital.^2^ For example, dedicated educational programs and standardized inpatient hyperacute stroke protocols have been shown to be effective in mitigating stroke onset‐to‐treatment delays at various institutions.^4^, ^5^, ^15^ However, there remain ongoing challenges that should be considered in the assessment and early recognition of stroke symptoms among inpatients after surgery, ventilated patients, those in the immediate perioperative period following a procedure under general anesthesia, and those with preexisting symptoms or delirium, particularly on nondedicated neurology wards.^2^ A slightly lengthy interval from neuroimaging to arterial puncture among patients with IHS could be explained by the possible need to transfer a proportion of EVT‐eligible patients from a primary stroke center to an EVT‐capable center. The lack of difference in the anesthesia mode employed between groups may be due to the standardized implementation of anesthesia protocols local to each institution, regardless of the possible underlying comorbidities.

The strengths of this study include its relatively large sample size of patients with IHS treated with EVT, the national coverage of a diverse range of hospitals and EVT‐capable neuroscience centers, and the high case ascertainment with consecutive patient enrollment. The accuracy and high‐quality data within the SSNAP database results from standardized case definitions and coding instructions, internal validation, audit trails, and regular data quality reports for all participating sites.^9^

There are several limitations in this study. First, due to its observational design, confounding by indication and selection bias may have influenced the results. The lack of the Alberta Stroke Program Early Computed Tomography Score, collateral status, and clot location, all key criteria in patient selection for EVT eligibility, limits the interpretation of these findings due to potential underlying selection biases. However, likely similar imaging criteria would have been employed for patient selection across both groups in various EVT centers. Second, there were some missing data for certain outcome measures, including the mRS at 6 months, while the mRS at 90 days is not collected in the registry. However, our primary outcome measured the mRS at hospital discharge (complete data in PSM cohort) and has been shown to correlate highly with functional outcomes at 3 months.^17^ It is possible that patients in the IHS groups could have had a longer hospital stay due to their comorbidities, though the lack of data on the duration of hospital stay precluded further comparison. Third, although there were some differences in between‐group baseline characteristics, the key variables were adjusted for in the PSM analysis. Fourth, the underlying reasons for the index hospital admission, further comorbidities such as underlying malignancy, and the proportion of transferred patients from a primary stroke center were not available in this registry, precluding further analyses. Fifth, the outcome measures, including the angiographic outcomes of vessel reperfusion, were self‐assessed rather than independently evaluated by a core laboratory. Finally, the assessment of treatment benefit of EVT in the IHS group is precluded due to the lack of comparison to a control group of patients that underwent best medical management only.

## Conclusions

In this national stroke registry, EVT in patients with IHS appears safe without any increase in sICH or mortality and has comparable functional outcomes to patients with COS. Continued efforts are required to improve the workflow in recognizing stroke symptoms and reduce the time‐to‐EVT treatment initiation for eligible patients with IHS.

## Sources of Funding

SSNAP is commissioned by the Health Quality Improvement Partnership and funded by National Health Service England and the Welsh Government. Martin A. James is supported by the National Institute for Health Research Applied Research Collaboration South West Peninsula. The views expressed in this publication are those of the author(s) and not necessarily those of the National Institute for Health Research or the Department of Health and Social Care. There was no funding for this study.

## Disclosures

Martin A. James has received lecture and consultancy fees from Medtronic. Thanh N. Nguyen reported research support from Medtronic and SVIN. Pervinder Bhogal reported travel support from Perflow and compensation from Cerenovus; Balt USA, LLC; Vesalio; Phenox Inc.; and Brainomix for consultant services. Hegoda Levansri Dilrukshan Makalanda reported compensation from Cerenovus, Balt USA, Perfuze, Stryker, Penumbra, Microvention, Neuroventures, and Brainomix for consultant services. No other relevant disclosures or competing interests declared by the remaining authors.
